# Aberrant Right Subclavian Artery With a Bicarotid Trunk: A Rare Cause of Stridor in Infants

**DOI:** 10.1002/ccr3.71310

**Published:** 2025-10-27

**Authors:** Mohammad Alashqar, Israa Salman, Shahd Saadeh, Seba Lubbadeh, Osayd Khasati

**Affiliations:** ^1^ Faculty of Medicine and Health Sciences An‐Najah National University Nablus Palestine; ^2^ Pediatrics and Neonatology Tulkarim Governmental Hospital Tulkarim Palestine

**Keywords:** cardiothoracic surgery, ear, nose and throat, radiology & imaging, respiratory medicine

## Abstract

The aberrant right subclavian artery represents a rare vascular anomaly that must be considered in the differential diagnosis of infants experiencing respiratory distress and feeding difficulties. Advanced imaging techniques, such as contrast‐enhanced computed tomography or magnetic resonance imaging, are essential for identifying this vascular anomaly and its anatomical course. Timely surgical intervention is critical as it can prevent severe complications and ultimately improve outcomes.

## 1 Introduction

**FIGURE 1:**
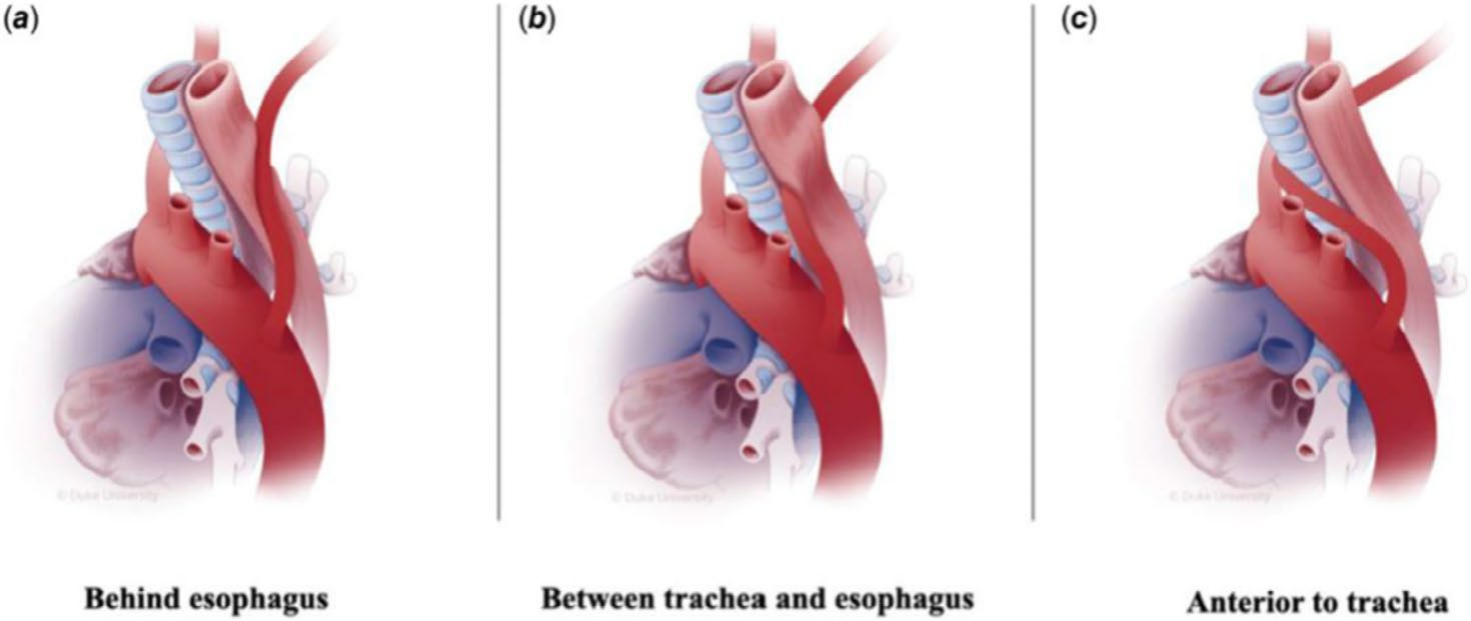
Anatomic configurations associated with aberrant right subclavian artery. In most cases, the aberrant right subclavian artery crosses behind the esophagus (A); however, it may rarely pass between the trachea and the esophagus (B) or anterior to the trachea (C).

Stridor encompasses a wide range of systemic causes, including both medical and surgical conditions. Common etiologies include laryngomalacia, congenital glottic webs, and tracheomalacia [1]. The aberrant right subclavian artery is a rare vascular anomaly that can also lead to respiratory distress in infants [2]. Its incidence is between 0.5% and 2% worldwide [2, 3]. In most cases, the aberrant right subclavian artery crosses behind the esophagus (Figure 1A); however, it may rarely pass between the trachea and the esophagus (Figure 1B) or anterior to the trachea (Figure 1C) [4, 5]. This leads to respiratory distress, stridor, and progressive dysphagia. Timely surgical intervention is critical; it can prevent severe complications and significantly improve outcomes. We report a case of an aberrant right subclavian artery in an infant that crosses behind the esophagus, leading to respiratory and digestive complications.

## 2 Case History/Examination

We present the case of a female term infant, aged 3 months and 15 days, who was delivered via normal vaginal delivery with a birth weight of 3200 g. The perinatal ultrasound was normal, but a detailed ultrasound was not performed. The infant was admitted to our hospital for progressive stridor that developed 10 days post‐delivery.

Upon admission to the neonatal intensive care unit for respiratory distress, the infant was intubated and ventilated for 10 days and subsequently transitioned to a high‐flow nasal cannula (Neonatal size) with a flow rate of 5 L/min. Her estimated daily nutritional requirements were approximately 480–576 mL of fluids and 320–384 kcal, based on standard recommendations of 150–180 mL/kg/day and 100–120 kcal/kg/day, respectively. There was no family history of immunodeficiency, surfactant deficiency, or underlying genetic disorders. On examination, bilateral air entry was good, and no murmurs were detected. However, inspiratory stridor was present, along with a contracture observed in the right hand. In addition, the infant had experienced difficulty gaining weight since birth.

## 3 Methods (Differential Diagnosis, Investigations and Treatment)

**FIGURE 2:**
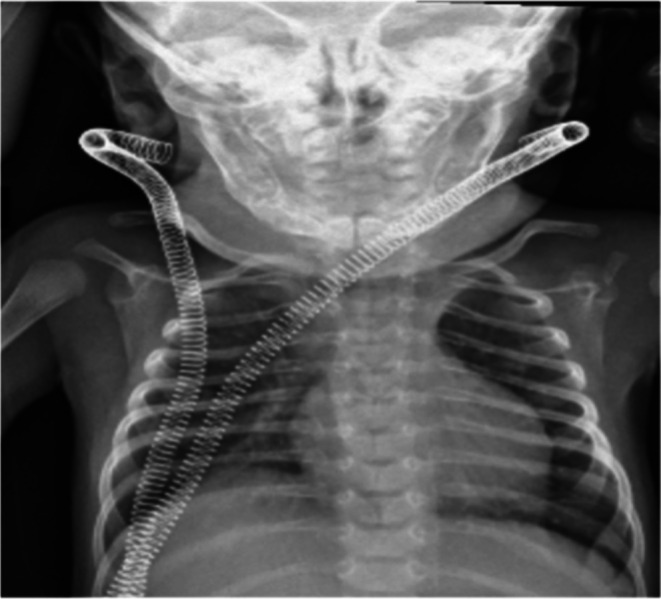
Chest X‐ray: normal.

Her initial complete blood count was normal. Because of her unstable respiratory state, she was intubated and ventilated for 10 days. A complete sepsis workup was performed, with negative results. An initial chest X‐ray showed normal findings (Figure 2).

**FIGURE 3:**
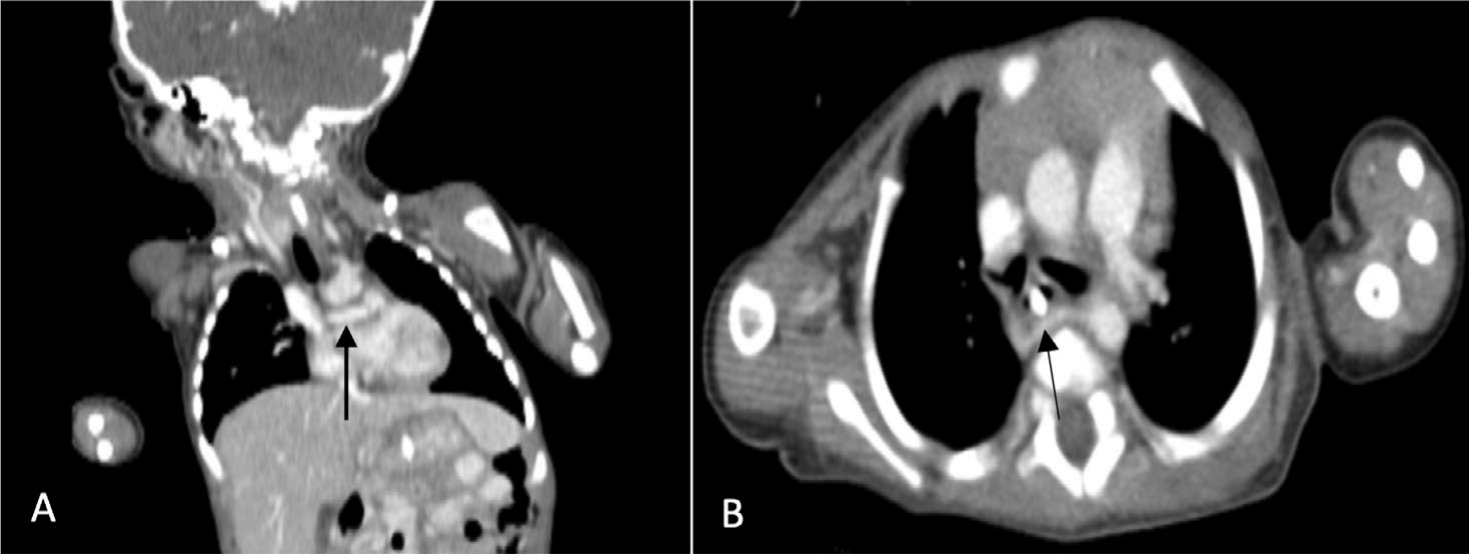
Neck and chest CT scan: white arrow indicates the retroesophageal course of the aberrant right subclavian artery (A, coronal; B, axial).

Laryngoscopy was performed, revealing severe laryngomalacia, which was managed conservatively with close monitoring, as her condition did not require surgical intervention. However, the stridor worsened over time, and as the infant began formula feeding, there was frequent vomiting, leading to a decrease in weight, as well as episodes of sweating, raising suspicion for an underlying anomaly. A contrast‐enhanced neck and chest CT scan was ordered using a low‐osmolar iodine‐based contrast agent, which demonstrated an aberrant right subclavian artery originating from the base of a left‐sided aortic arch and coursing behind the esophagus (Figure 3).

**FIGURE 4:**
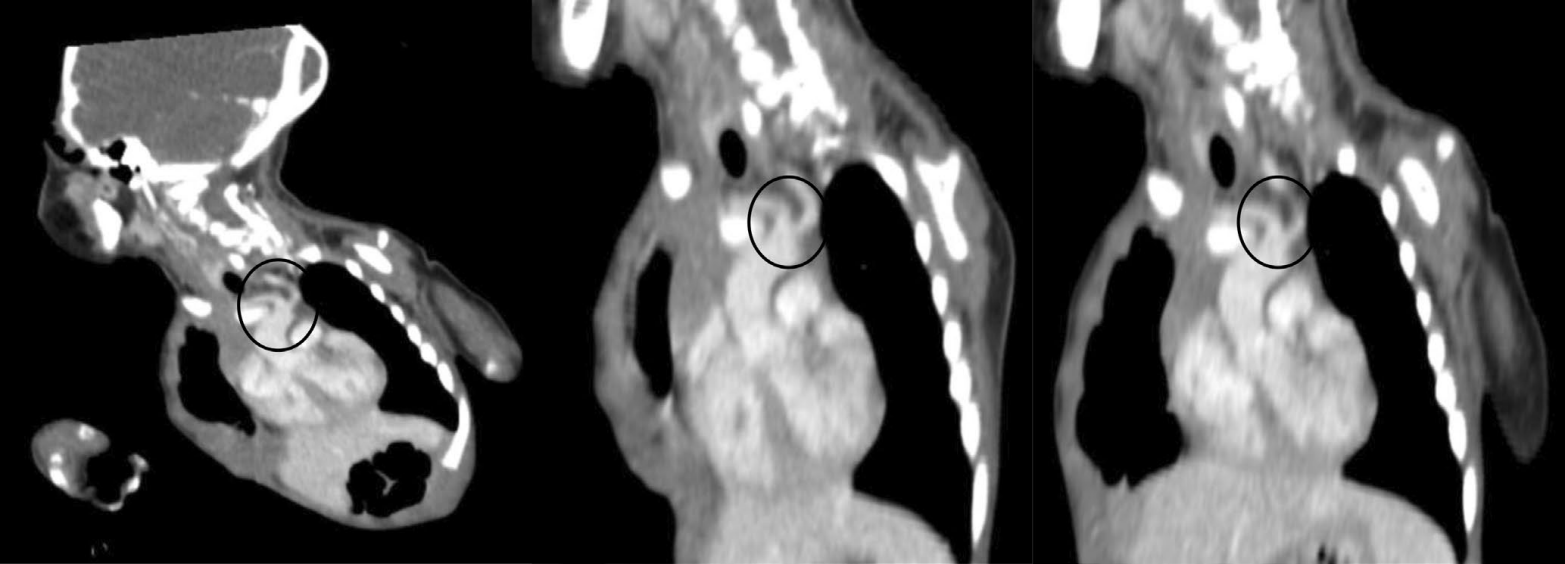
These are reformatted images illustrating the origin of both common carotid arteries (CCAs).

The heart and kidney appeared normal. However, the right vertebral artery originates from the aberrant right subclavian artery, while the left vertebral artery originates directly from the aorta (the fourth branch after the aberrant right subclavian artery). In addition, both carotid arteries share a trunk, a condition known as a bicarotid trunk anomaly (Figure 4). No evidence of a Kommerell diverticulum was found in this case. The cardiothoracic department was consulted, and she was prepared to undergo surgical resection and was referred to a specialized center for management.

## 4 Conclusion and Results

This case highlights the necessity to recognize an aberrant right subclavian artery as a possible cause of respiratory distress in infants who present with stridor. Given its unusual anatomical path and the possibility of airway constriction, early detection with imaging, particularly contrast‐enhanced computed tomography, is critical for effective treatment. Our findings highlight the importance of comprehensive evaluation in patients with persistent respiratory symptoms in order to avoid misdiagnosis and ensure timely intervention. Infants with an aberrant right subclavian artery can improve their respiratory problems significantly with adequate surgical correction, emphasizing the need for early recognition and detection of this vascular abnormality in pediatric surgery.

## 5 Discussion

### 5.1 Clinical Significance of Stridor

Stridor is a high‐pitched respiratory sound that occurs during inspiratory, expiratory, or both and indicates an airway anomaly [1]. Stridor can occur alone or in conjunction with other indicators of increased work of breathing, such as tachypnea, nasal flaring, chest retractions, or grunting [6]. The characteristic sound of stridor can be broadly classified as inspiratory, expiratory, or biphasic [7]. Inspiratory stridor indicates supraglottic pathologic situations, biphasic stridor occurs in glottic or subglottic pathologic conditions, and expiratory stridor is more common in tracheal pathologic conditions [8]. These characteristics are broad, and they should not be used to rule out pathological disorders. Instead, they help to narrow the scope of a clinician's evaluation.

### 5.2 Embryological Background

The aortic arch gives rise to 3 main vessels: the brachiocephalic trunk, which divides into the right subclavian and right common carotid arteries, the left common carotid, and the left subclavian [9]. These are major elastic arteries and help to stabilize blood flow. During embryonic development, the subclavian arteries come from the aortic arches, which start to form in the 4th week of intrauterine life. The right subclavian artery comes from the 4th right aortic arch, the dorsal aorta, and the 7th right intersegmental artery, while the left subclavian comes from the 7th left intersegmental artery [10].

### 5.3 Anatomical Variations and Classification

**FIGURE 5:**
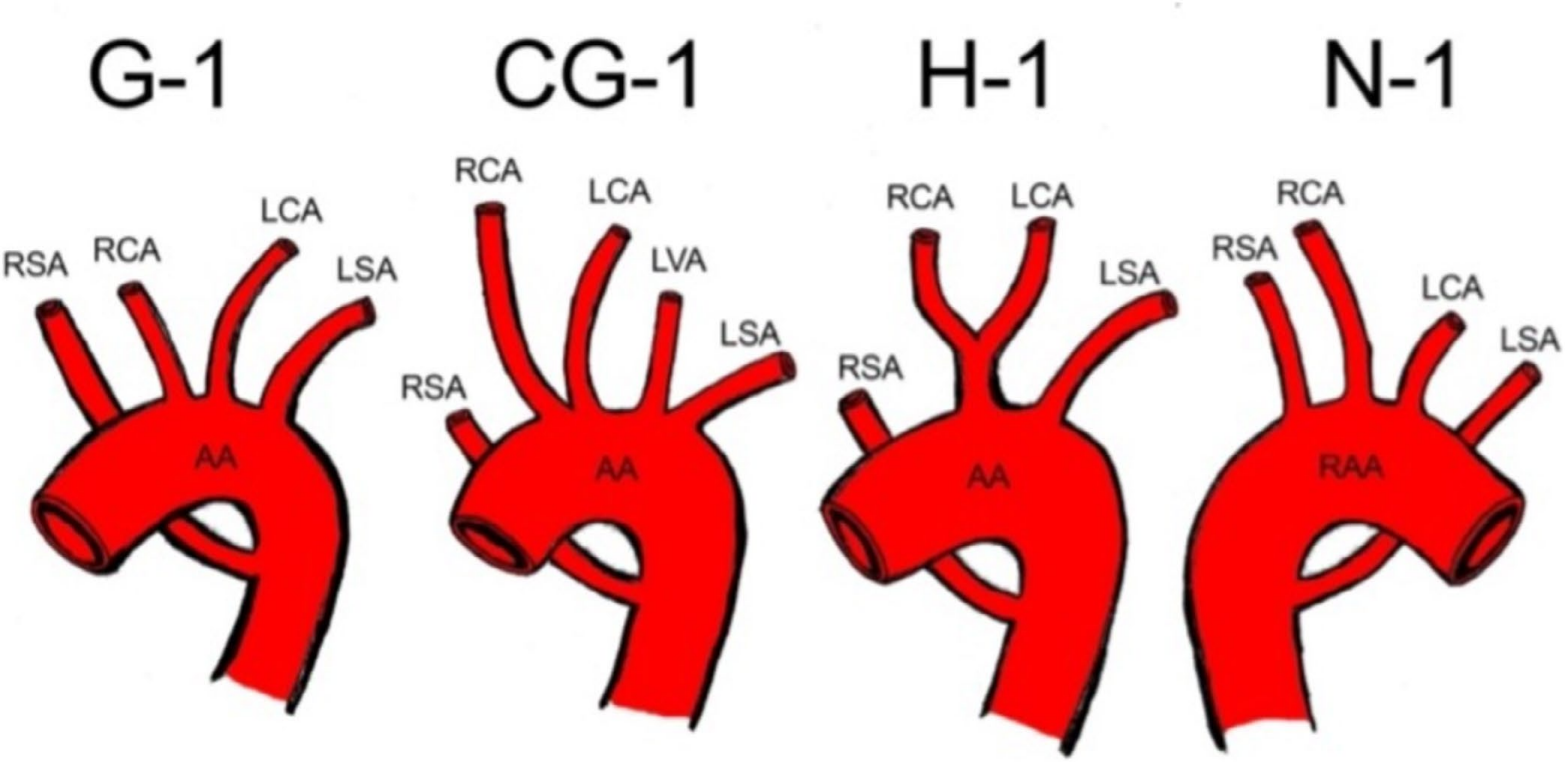
Schematic drawing based on the Adachi–Williams classification of right subclavian artery anomalies. AA, aortic arch; LCA, left common carotid artery; LSA, left subclavian artery; LVA, left vertebral artery; RAA, right aortic arch; RCA, right common carotid artery; RSA, right subclavian artery.

In rare cases, the primitive aortas and aortic arches can develop abnormally and result in an aberrant right subclavian artery. This happens when the distal part of the right dorsal aorta and the 7th intersegmental artery contribute to its development, and the 4th right aortic arch and the proximal part of the dorsal aorta, which would be involved in its formation, are obliterated [11]. Subsequently, the aberrant right subclavian artery comes off as the last branch of the aortic arch. The Adachi and Willians classification divides the aberrant right subclavian artery into four basic types [12] (Figure 5):

- Type G‐1: aberrant right subclavian artery, originates from the distal end of the aortic arch as its last branch.
- Type CG‐1: aberrant right subclavian artery is in the same anatomic position as in type G, but the left vertebral artery comes off the aortic arch as an additional branch, as observed in this case.
- Type H‐1: aberrant right subclavian artery is the last branch of the aortic arch.
- Type N‐1: in this type the distribution is the mirror image of type G, the aortic arch is on the right and a left subclavian imitates what would be an arteria lusoria.

### 5.4 Clinical Associations and Complications

Vascular ring malformations are rare, with one of the most common being an aberrant subclavian artery. It affects approximately 0.7%–2% of the population with a left aortic arch and 0.04%–0.4% with a right aortic arch [7, 8]. The incidence between 0.5% and 2% worldwide has been found for the aberrant right subclavian artery [2]. In 80% of situations, an aberrant subclavian artery passes behind the esophagus [13]. In 15% of cases, it crosses between the trachea and the esophagus, while in 5%, it is located anterior to the trachea [14]. In the majority of cases, the aberrant right subclavian artery causes no symptoms [11]. It can coexist with a bicarotid trunk as in our case; however, this is extremely rare, with an estimated prevalence of < 0.05% [15].

The presence of an aberrant right subclavian artery is frequently associated with other anatomical abnormalities. One of these is a non‐recurrent laryngeal nerve, which was detected alongside an aberrant right subclavian artery in 86.7% [16]. If the aberrant path is not identified in advance, it might cause surgical [17, 18] and post‐surgical iatrogenic consequences, such as vocal cord paralysis. 25% to 37% of patients have congenital cardiac issues, such as conotruncal anomalies or other chromosomal abnormalities [2].

### 5.5 Diagnostic Approaches

Contrast‐enhanced CT and cardiac magnetic resonance imaging are the preferred diagnostic modalities for detecting suspected vascular rings. They provide identification of the abnormality, its location, branching pattern, and arch dominance, as well as the assessment of the degree of airway and esophageal compression [2]. Three‐dimensional reconstruction is useful for planning surgical interventions.

### 5.6 Treatment Outcomes

The treatment for an aberrant right subclavian artery may differ based on the patient's age and any additional vascular malformations present [19]. However, simple vascular ring repair in infants results in 92% remission from respiratory symptoms 1 year later [2]. Although a small number of patients may require reoperation, proper preoperative imaging and initial surgery can reduce this risk.

## Author Contributions

**Mohammad Alashqar:** software, validation, writing – original draft, writing – review and editing. **Seba Lubbadeh:** writing – original draft. **Israa Salman:** supervision. **Shahd Saadeh:** writing – original draft. **Osayd Khasati:** writing – original draft.

## Ethics Statement

Informed consent was obtained from the patient's parents. Our institution does not require ethical approval for case reports.

## Consent

Written informed consent was obtained from the patient's parents for publication of this case report and the accompanying images.

## Conflicts of Interest

The authors declare no conflicts of interest.

## Data Availability

The data supporting the findings of this case report is not available due to privacy and confidentiality restrictions.
